# Freeze-Dried Porous Collagen Scaffolds for the Repair of Volumetric Muscle Loss Injuries

**DOI:** 10.1021/acsbiomaterials.4c01601

**Published:** 2025-02-05

**Authors:** Ivan M. Basurto, Geshani C. Bandara, Ryann D. Boudreau, Sydney B. Shriver, Samir A. Muhammad, George J. Christ, Steven R. Caliari

**Affiliations:** ^†^Department of Biomedical Engineering, ^‡^Department of Chemical Engineering, ^§^Department of Orthopedic Surgery, University of Virginia, Charlottesville, Virginia 22903, United States

**Keywords:** volumetric muscle losss, collagen-glycosaminoglycan, CG-polypyrrole (PPy)

## Abstract

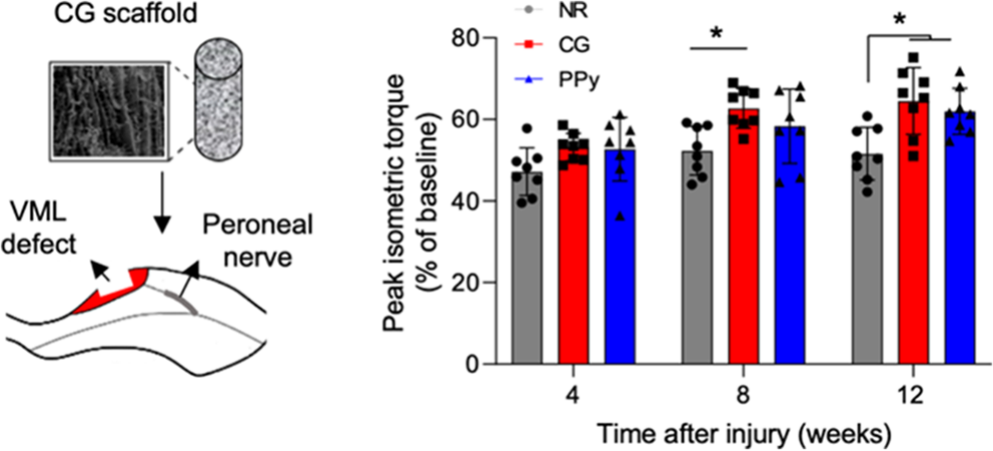

Volumetric muscle loss (VML) injuries are characterized by the traumatic loss of skeletal muscle, resulting in permanent damage to both tissue architecture and electrical excitability. To address this challenge, we previously developed a three-dimensional (3D) aligned collagen-glycosaminoglycan (CG) scaffold platform that supported *in vitro* myotube alignment and maturation. In this work, we assessed the ability of CG scaffolds to facilitate functional muscle recovery in a rat tibialis anterior (TA) model of VML. Functional muscle recovery was assessed following implantation of either nonconductive CG or electrically conductive CG-polypyrrole (PPy) scaffolds at 4, 8, and 12 weeks postinjury by *in vivo* electrical stimulation of the peroneal nerve. After 12 weeks, scaffold-treated muscles produced maximum isometric torque that was significantly greater than nontreated tissues. Histological analysis further supported these reparative outcomes with evidence of regenerating muscle fibers at the material–tissue interface in scaffold-treated tissues that were not observed in nonrepaired muscles. Scaffold-treated muscles possessed higher numbers of M1 and M2 macrophages at the injury, while conductive CG-PPy scaffold-treated muscles showed significantly higher levels of neovascularization as indicated by the presence of pericytes and endothelial cells, suggesting a persistent wound repair response not observed in nontreated tissues. Finally, only tissues treated with nonconductive CG scaffolds displayed neurofilament staining similar to native muscle, further corroborating isometric contraction data. Together, these findings show that both conductive and nonconductive CG scaffolds can facilitate improved skeletal muscle function and endogenous cellular repair, highlighting their potential use as therapeutics for VML injuries.

## 1 Introduction

Volumetric muscle loss (VML) injuries result in an aberrant wound healing response that is characterized by chronic inflammation, persistent activation of myofibroblasts, excessive collagen deposition, and ultimately a loss of muscle mass and function.^1−3^ These injuries are defined by the removal of a large volume of muscle tissue and the surrounding extracellular matrix (ECM) that is typically due to high-energy trauma, such as blast injuries and vehicle accidents, surgical ablations, or myopathies.^4^ The current clinical standard for the treatment of VML injuries is autologous tissue transfer. However, this process is time-intensive and expensive and results in variable levels of functional success.^5^ Furthermore, donor site morbidity as well as heterogeneity between patients and injuries has made effective treatment an enduring challenge.^6,7^ As a result, there is a clear need to design novel therapeutic approaches for VML injuries.

To address the treatment limitations of VML, researchers have focused on the use of biomaterial systems to support endogenous repair mechanisms. These materials have varied widely to include natural and synthetic polymers augmented with growth factors, cells, and other biophysical cues. The considerable diversity of biomaterials used for skeletal muscle tissue engineering has been reviewed extensively elsewhere.^8−12^ Careful design of biomaterial systems is critical to support improved regenerative outcomes because subtle changes to material properties can either facilitate regeneration or drive suboptimal pathological outcomes.^13^ Two biophysical features that are characteristic of healthy skeletal muscle function are the high level of structural organization and the electrochemical excitability, which enable efficient contraction, force generation, and locomotion. This is particularly relevant following VML where the ECM becomes disorganized, hindering cell-mediated repair,^14^ and a prolonged loss of electrical stimuli results in continual muscle atrophy.^15−17^

In an effort to recreate these key biophysical features, we have previously developed three-dimensional (3D) aligned collagen-glycosaminoglycan (CG) scaffolds for skeletal muscle tissue engineering.^18,19^ CG scaffolds have a rich history of clinical use and were previously FDA-approved for skin regeneration.^20,21^ Building on this platform, our previous work used a directional freezing process that resulted in aligned collagen struts reminiscent of healthy skeletal muscle ECM following lyophilization.^22^ To recapitulate the electrical properties of skeletal muscle, we also demonstrated the ability to synthesize and incorporate electrically conductive polypyrrole (PPy) particles into our CG suspension prior to freeze-drying. The inclusion of PPy resulted in a 5-fold increase (1.42 ± 0.18 versus 0.27 ± 0.04 mS m^–1^) in material conductivity that did not detrimentally impact myoblast metabolic activity.^18^ Moreover, our PPy-doped scaffolds supported 3D cytoskeletal alignment along the scaffold’s backbone and promoted enhanced myoblast maturation measured by myosin heavy chain (MHC) expression.^18^ As a logical extension of our prior work, we aim to explore how 3D-aligned collagen scaffolds, in both nonconductive and electrically conductive forms, influence skeletal muscle repair in an *in vivo* rat VML injury model.

## 2 Materials and Methods

### 2.1 Polypyrrole (PPy) Synthesis

PPy nanoparticles were synthesized as previously described.^18^ Briefly, 2 g of pyrrole monomer was reacted with 72 mmol of FeCl_3_ using vigorous mixing for 24 h under ambient conditions. The resulting black precipitate was washed repeatedly with water and filtered using vacuum filtration. The PPy powder was then dried overnight and passed through a 325 mesh (45 μm) screen. Fourier-transform infrared (FTIR) spectroscopy was then used to confirm the chemical structure of the PPy particles.

### 2.2 Scaffold Fabrication

Nonconductive collagen-glycosaminoglycan (CG) scaffolds were fabricated by homogenizing a suspension of 1.5 wt % microfibrillar type I collagen from bovine Achilles tendon and 0.133 wt % chondroitin sulfate derived from shark cartilage in 0.05 M acetic acid. The suspension was prepared in a recirculating chiller maintained at 4 °C to prevent collagen denaturation. Conductive PPy-doped (CG-PPy) scaffolds were created by mixing PPy nanoparticles (0.5 wt %) into the collagen/chondroitin sulfate suspension via vortexing. All scaffolds were fabricated via directional lyophilization using a thermally mismatched mold^18,23^ in a VirTis Genesis pilot scale freeze-dryer. Following lyophilization scaffolds were dehydrothermally cross-linked at 105 °C for 24 h.

### 2.3 Scaffold Hydration and Cross-Linking

The resulting scaffold cylinders (∼15 mm height, ∼6 mm diameter) were hydrated in 70% ethanol for 30 min before being transferred to phosphate-buffered saline (PBS). The scaffolds were then chemically cross-linked using 1-ethyl-3-(−3-(dimethylamino)propyl) carbodiimide hydrochloride (EDC) and *N*-hydroxysulfosuccinimide (NHS) at a molar ratio of 5:2:1 EDC/NHS/COOH where COOH is the carboxylic acid content of the collagen. EDC/NHS-mediated cross-linking facilitates the covalent reaction of collagen primary amines with carboxylic acids to improve scaffold mechanical integrity.^24^ CG scaffolds were incubated in sterile-filtered EDC/NHS solution for 50 min under moderate shaking before being washed twice with PBS. The scaffolds were then transferred to 70% ethanol to sterilize overnight before being washed repeatedly with sterile PBS. All scaffolds were stored at 4 °C in sterile PBS until the time of surgery.

### 2.4 Animal Care

This study was conducted in compliance with the Animal Welfare Act and the Implementing Animal Welfare Regulations and in accordance with the principles of the Guide for the Care and Use of Laboratory Animals. The University of Virginia Animal Care and Use Committee approved all animal procedures. A total of 24 male Lewis rats (Charles River Laboratories) age-matched to 11 weeks weighing 312.7 ± 24.9 g were purchased and individually housed in a vivarium accredited by the American Association for the Accreditation of Laboratory Animal Care and provided with food and water *ad libitum*.

### 2.5 Surgical Procedures

The VML injury model was created using a previously established tibialis anterior (TA) injury model.^25,26^ Rats were randomly designated to three different experimental groups: no repair (NR; *n* = 8), nonconductive CG scaffolds (CG; *n* = 8), and conductive PPy-doped CG scaffolds (CG-PPy; *n* = 8). To create the surgical defect, rats were anesthetized via isoflurane, and the surgical site was aseptically prepared by repeated washes with alcohol and iodine. A longitudinal skin incision was made on the anterior side of the lower left leg to expose the anterior crura muscles. The skin was then separated from the underlying fascia by using surgical scissors. The fascia were then separated to expose the underlying musculature. Next, the extensor digitorum longus (EDL) and extensor hallucis longus (EHL) muscles were surgically ablated to avoid compensatory hypertrophy of synergistic muscles involved in dorsiflexion during later *in vivo* functional assessment.^25^ The VML defect size was approximated from a validated linear regression to estimate TA muscle mass from rat body weight.^25,26^ The injury was created by surgically resecting approximately 20% of the TA muscle weight from the middle third of the TA muscle. Following the creation of the injury, scaffolds (8 mm diameter ×15 mm length cylinders, cut in half longitudinally) were sutured into the defect using 6–0 Vicryl. The fascia were then sutured back in place using 6–0 Vicryl sutures, and the skin was closed with 5–0 Prolene using interrupted sutures. Skin glue was applied over the top of the Prolene sutures to avoid reopening of the injury. Following surgery, animal health was monitored daily, and skin sutures were removed after 14 days.

### 2.6 *In Vivo* Functional Testing

Isometric force testing was performed on animals at 1 week prior to surgery and at 4, 8, and 12 weeks postsurgery to quantify the functional deficit created by the surgical defect and track recovery over time. *In vivo* force testing was conducted using an established protocol in which isometric torque is produced as a function of stimulation frequency (1–200 Hz).^27^ Rats were anesthetized via isoflurane, and the left hindlimb was aseptically prepared by repeated washes with alcohol and iodine. The foot was secured against a force transducer foot plate, ensuring that the heel was flush with the bottom of the plate. The knee joint was then stabilized and positioned so that the foot and tibia were at a 90° angle. Force testing was performed by stimulation of the peroneal nerve with platinum needle electrodes. Electrodes were placed in the posterior compartment of the lower leg along either side of the peroneal nerve, and muscle length was adjusted until maximal twitch force was produced. The contractile force of the anterior crura muscles was then assessed by measuring peak isometric tetanic force production as dorsiflexion occurred. Torques at all time points were normalized to the body weight of the animal at the time of stimulation.

### 2.7 Histology Analysis

Following functional testing, all retrieved muscles were photographed before being frozen for further processing. The TA muscle was cut in half cross-sectionally, and the proximal portion was embedded in OCT before being flash frozen in liquid nitrogen-cooled isopentane. Distal muscle sections were cut in half longitudinally and embedded in OCT for cryosectioning. Distal sections were used for the analysis of muscle innervation while proximal tissue sections were used to quantify muscle fiber cross-sectional area (FCSA), macrophage infiltration, and neovascularization. All muscle samples were then cryosectioned (8 μm-thick slices) and placed on glass slides. Hematoxylin and eosin (H&E) stains were administered using conventional techniques to analyze tissue morphology, muscle repair, and fibrotic response for at least 3 muscles per experimental group. Stained muscle sections were then visualized using a Leica Thunder imaging system at 10× magnification.

### 2.8 Immunohistochemical (IHC) Analysis

Unstained OCT-embedded slides were first subjected to an antigen retrieval protocol (H-3301; Vector Laboratories) in preparation for antibody staining. To reduce the autofluorescence of muscle tissue, samples were treated with 0.3% Sudan black solution for 10 min prior to blocking for 2 h (Dako Blocking Solution X0909; Agilent Technologies, Santa Clara, CA). Immunohistochemical staining was then performed using antibodies to detect laminin (dilution 1:200, ab11575; Abcam), CD68 (1:100, MCA341R; Bio-Rad) and CD163 (1:400, ab182422; Abcam), CD31 (1:250; Novus Biologicals NB100–2284) and α-smooth muscle actin (αSMA, conjugated to 488 fluorochrome, F3777, 1:250; Sigma-Aldrich), or neurofilament 200 (NF200, antichicken, 1:1000; EnCor CPCA-NF-H) overnight at 4 °C. Next, samples were incubated for 2 h at ambient temperature with one or more of the following secondary antibodies: Alexa Fluor 647 Fab 2 fragment goat antirabbit (1:500), Alexa Fluor 488 goat antichicken (1:600), Alexa Fluor 488 goat antimouse (1:400), and/or Alexa Fluor 488 goat antirabbit (1:400). Finally, slides were stained with DAPI (1:1000) before being stored in a light-protected environment at −20 °C until imaging.

All samples were imaged on a Leica inverted confocal microscope using a 10× objective across the entire TA muscle section (*n* = 3 muscles for each group). For quantification of macrophage infiltration and polarization, image analysis was conducted at the injury site, defined as a 4 mm × 2 mm region at the VML injury. A custom MATLAB code was then used to quantify the number of M1 (CD68^+^/CD163^–^) and M2 (CD163^+^) macrophages throughout this region. CD31 and αSMA-labeled structures were quantified using CellProfiler. For neurofilament-stained images, NF200^+^ structures were quantified across the entire longitudinal muscle sections using ImageJ.

### 2.9 Muscle Fiber Quantification

Muscle sections stained with laminin were used to visualize the ECM and quantify fiber cross-sectional area (FCSA), minimum Feret diameter (the measure of an object size along its minimum axis), and total fiber count. Laminin-stained muscle sections were visualized using a Leica DMi8 inverted confocal microscope with a 5× objective. Image postprocessing was conducted using ImageJ. Muscle fiber quantification was conducted using a publicly available semiautomatic muscle analysis using segmentation of histology (SMASH) software.^28^ Briefly, individual muscle fibers were outlined using SMASH’s built-in segmentation algorithm based on laminin staining. The segmentation output was visually inspected, and incomplete or incorrect segmentation was manually adjusted. Following correct segmentation, the number of muscle fibers and FCSA were calculated by the software across the whole TA muscle section. To gain a more thorough understanding of the quality of muscle repair, SMASH analysis was repeated at the VML injury site (4 mm × 2 mm region at the VML injury) to quantify the minimum Feret diameter and FCSA across experimental groups. The analysis was completed in triplicate for each experimental group.

### 2.10 Statistical Analysis

Data are presented as means and their standard deviations (SDs) unless otherwise indicated. Histological, immunohistochemical, and muscle fiber quantification was conducted for *n* = 3 muscles per group. Data normality distribution was evaluated using the D’Agostino and Pearson test. Kruskal–Wallis with Dunn’s multiple comparisons tests were performed for data that were not normally distributed. Functional data were statistically analyzed using a paired two-way analysis of variance (ANOVA) while immunohistochemical staining was evaluated using a one-way ANOVA. Upon finding any statistically significant differences via ANOVA, posthoc multiple comparison tests of parameters of interest were performed using Tukey’s HSD or Dunnett’s multiple comparisons test. These statistical analyses were conducted using GraphPad Prism 9.0. *P* values <0.05 were considered statistically significant.

## 3 Results

### 3.1 Creation of Rat TA VML Injury and Scaffold Delivery

**Figure 1:**
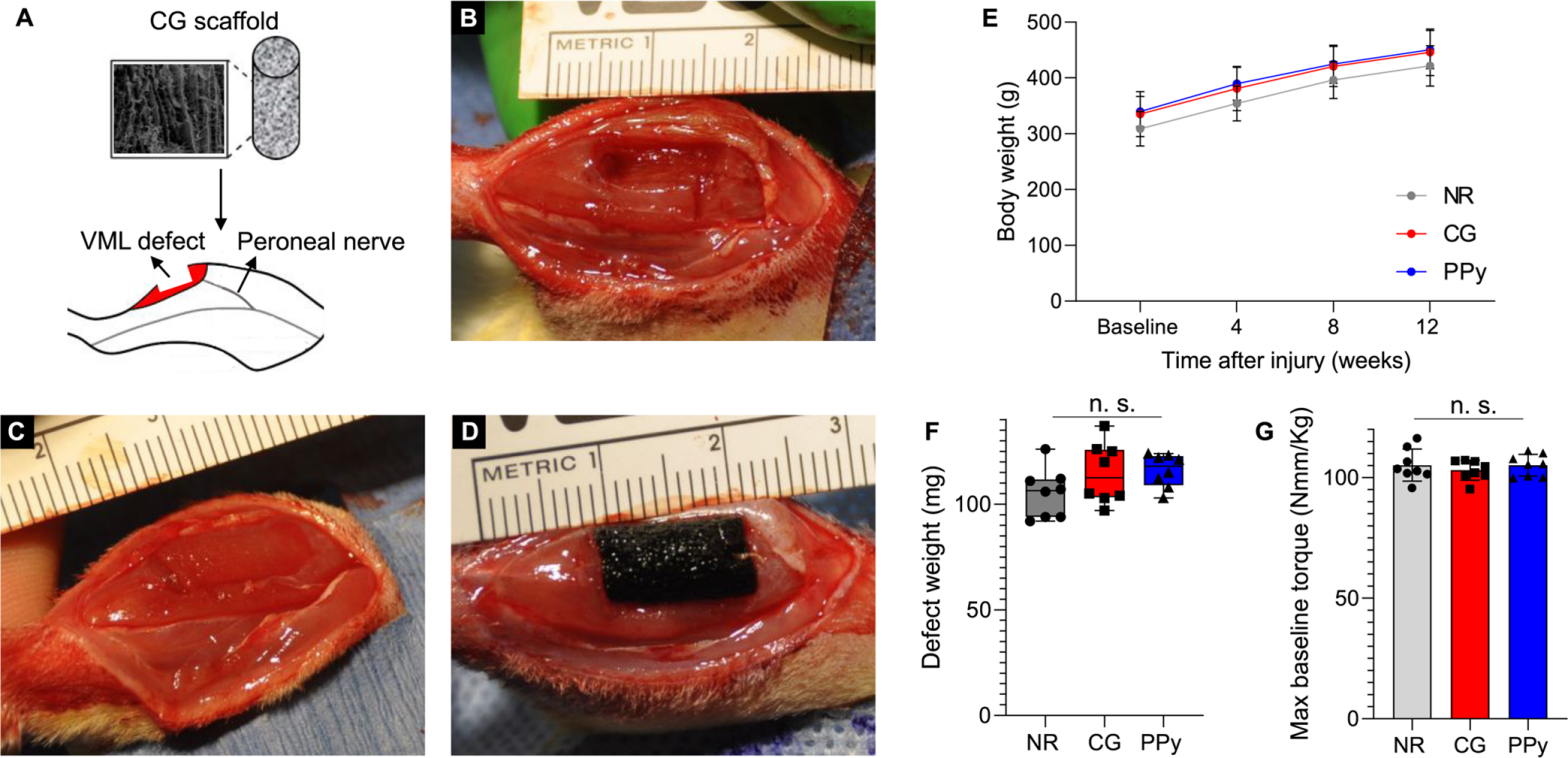
CG scaffolds were surgically implanted into a tibialis anterior (TA) model of VML injury. (A) Schematic representation of scaffold delivery into the TA VML injury model. Representative images of (B) no repair, (C) nonconductive CG scaffold, and (D) PPy-doped CG scaffold-treated muscles. (E) Animal body weight 1 week prior to surgery and at 4, 8, and 12 weeks post-VML injury, corresponding to functional testing time points. (F) Weight of defects created for no repair, nonconductive CG scaffold, and conductive PPy-doped CG scaffold (NR, CG, and PPy, respectively) experimental groups. (G) Maximum baseline torque generation normalized to animal body weight preinjury. Data are presented as Mean ± SD (panels E, G) while panel (F) data are presented as box plots of interquartile range (line: median) with whiskers showing minimum and maximum values. *n*.*s*.: no statistically significant differences. *n* = 8 animals per experimental group.

CG scaffolds, either with or without PPy, were successfully fabricated and mechanically characterized prior to surgical implantation, with no significant differences in stiffness observed between the nonconductive CG or conductive PPy-doped CG scaffolds (Figure S1). Scaffolds were surgically delivered into an established rat tibialis anterior (TA) model of a volumetric muscle loss (VML) injury. The weight of excised muscle was statistically similar across experimental groups, indicating the reproducible creation of VML injuries. Three experimental groups were investigated: No repair (NR: 105.3 ± 11.6 mg, *n* = 8) and implantation of either nonconductive CG scaffolds (CG: 114.6 ± 14.2 mg, *n* = 8) or conductive PPy-doped CG scaffolds (PPy: 116.0 ± 7.8 mg, *n* = 8) (Figure 1). Following surgical creation of the defect, scaffolds were cut to the dimensions of the injury and sutured into place. No deaths occurred following the procedure, and all animals recovered with no visual signs of infection or additional treatment. Moreover, animal body weights were statistically similar at the time of surgery and underwent comparable increases over the course of the study.

### 3.2 Evaluation of TA Functional Recovery Post-VML Injury

**Figure 2:**
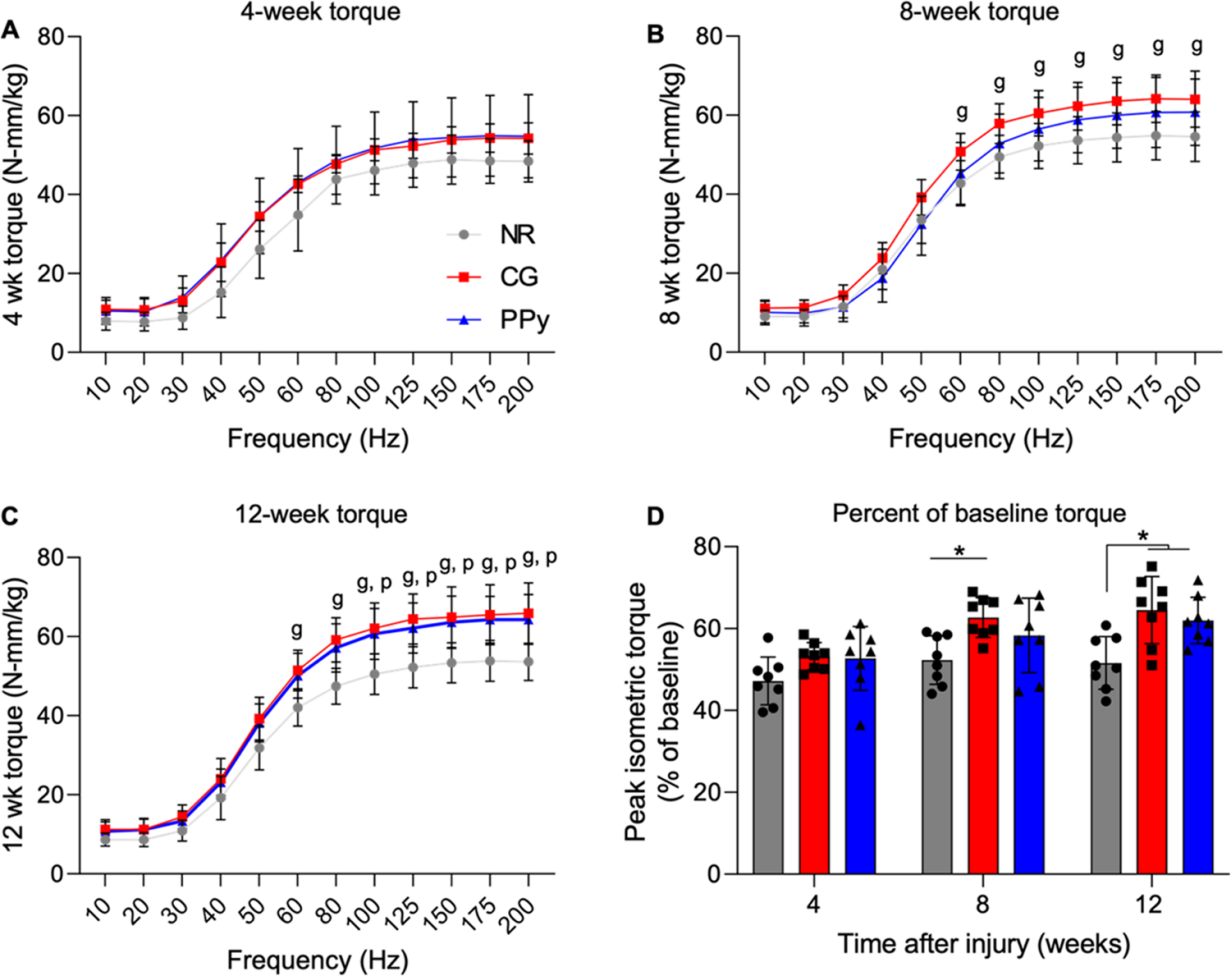
Both conductive and nonconductive scaffolds supported improved functional muscle recovery at 12 weeks post-VML. (A) Torque *vs* frequency curves at 4 weeks postinjury show a roughly 50% drop in muscle force from baseline values. (B) At 8 weeks post-VML, nonconductive scaffold-treated muscles showed significantly increased torque production at higher stimulation frequencies. (C) At 12 weeks postinjury, conductive CG-PPy and nonconductive CG scaffold-treated tissues produced significantly more force in response to higher stimulation frequencies. (D) Peak isometric contraction normalized to baseline values showed that both CG-PPy and CG scaffolds facilitated increased functional recovery compared to nontreated tissues. Data are presented as Mean ± SD. Statistically significant differences compared to the no repair group are denoted by *g* (CG) and *p* (PPy). * *P* < 0.05. *n* = 8 animals per experimental group.

Functional muscle contraction was assessed prior to surgery and at 4, 8, and 12 weeks postsurgery by *in vivo* stimulation of the TA muscle. Briefly, the animal’s left hindlimb was affixed to a force transducer, and electrical probes were inserted along the peroneal nerve. Isometric contractile force in response to direct muscle electrical stimulation was recorded over a range of frequencies (1–200 Hz). Force production was reported as torque normalized to body weight at each time point (N-mm kg^–1^ of body weight) to account for increases in force production due to animal growth. There were no statistical differences in the maximum baseline isometric torque between experimental groups prior to surgery. Additionally, baseline values reflect our previously published data using similar methods.^29−31^ At 4 weeks post-VML, there were no statistical differences in torque production among the treatment groups (Figure 2). NR and scaffold-treated muscles produced a maximum isometric torque that was roughly 50% of baseline values (NR: 49.5 ± 5.7 N-mm kg^–1^, CG: 54.8 ± 3.8 N-mm kg^–1^, PPy: 55.3 ± 7.8 N-mm kg^–1^) and was similar to previously published results.^29−31^ At 8 weeks following injury, nonconductive CG scaffold-treated animals showed significantly improved isometric contraction at higher stimulation frequencies (60–200 Hz) compared to NR animals. When peak isometric torque was normalized to baseline values, CG scaffold-treated animals produced significantly greater force (62.7 ± 4.9%) than NR animals (52.3 ± 5.9%). A similar trend was observed in conductive CG-PPy-treated animals although these results were not statistically significant. Finally, at 12 weeks postinjury, both conductive CG-PPy and nonconductive CG scaffold-treated animals produced significantly greater isometric torque at higher stimulation frequencies (100–200 and 60–200 Hz, respectively) compared to nontreated animals. This trend was conserved when peak isometric torque was normalized to baseline values (NR: 51.6 ± 6.4; CG: 64.5 ± 8.2; PPy: 62.0 ± 5.7%) indicating superior functional muscle recovery by the addition of collagen scaffolds compared to nontreated animals.

### 3.3 Gross Tissue Morphology

**Figure 3:**
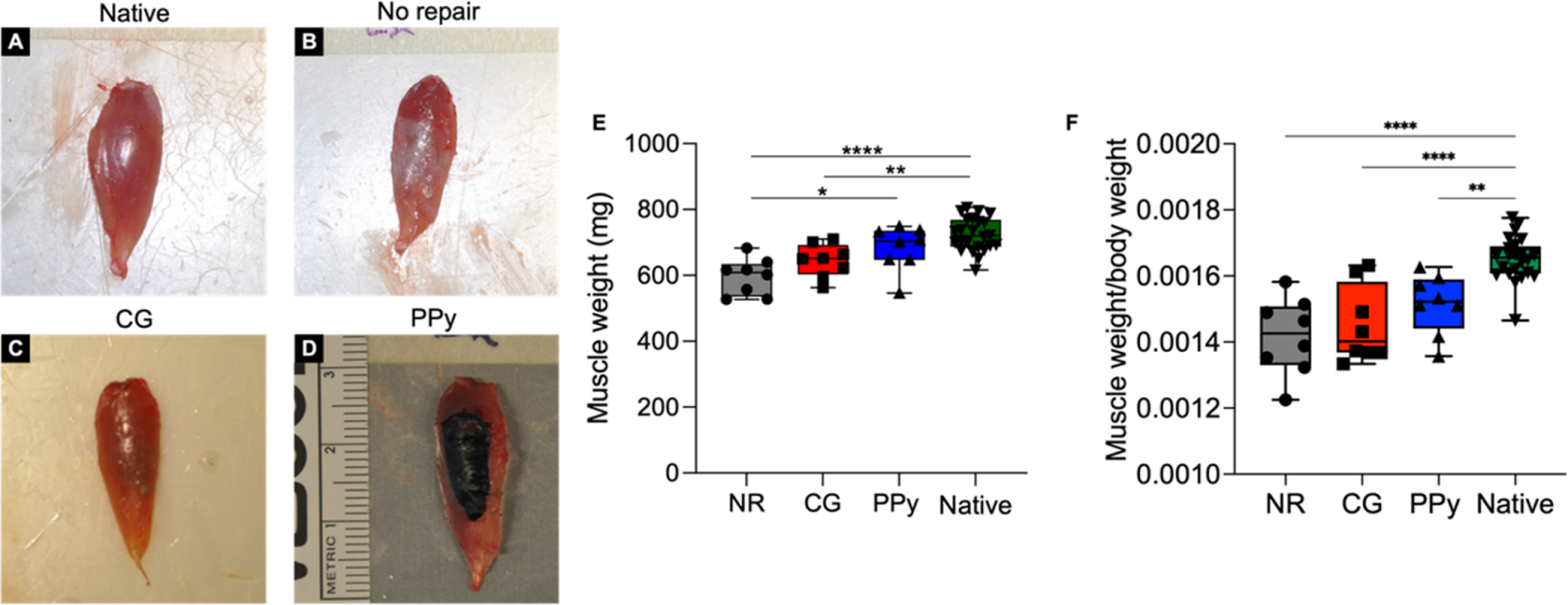
Gross tissue morphology at 12 weeks post-VML indicates a disparate biological response to therapeutic intervention. (A) Representative image of uninjured native tissue. (B) Nonrepaired muscles possessed a layer of fibrotic and fatty tissue that surrounded the VML injury area. (C) CG scaffold-treated muscles displayed lower levels of fibrosis, but some ECM deposition was observed. (D) PPy particles remained localized to the VML defect although the collagen scaffold appeared degraded. (E) Muscle weight at the time of explant shows that NR and CG scaffold-treated tissues were significantly lighter than native muscle. (F) When normalized to animal body weight, explanted muscle weight was significantly reduced for all experimental groups compared to native muscle. Data are presented as box plots of interquartile range (line: median) with whiskers showing minimum and maximum values. * *P* < 0.05, ** *P* < 0.01, **** *P* < 0.0001. *n* = 8 muscles per experimental group and *n* = 24 for the native contralateral control.

Following functional testing at 12 weeks post-VML, the TA of both injury and contralateral limbs was surgically explanted for morphological and histological analyses. Nonrepaired muscles possessed a visible layer of fibrotic tissue and fatty deposition over the VML injury area (Figure 3). Additionally, visual inspection of nontreated muscles showed a convex surface at the defect compared to native contralateral control tissues. In contrast, nonconductive CG scaffold-treated muscles contained lower levels of ECM deposition, although some fibrotic tissue remained. Importantly, a clear discontinuity between the muscle tissue and implanted scaffold was not observed, indicating some degradation and integration of the CG scaffold. Residual PPy particles were clearly observed at the VML injury site in conductive scaffold-treated muscles 12 weeks postinjury. While the particles remained, the collagen scaffold backbone appeared to be fully degraded as in the CG scaffold-treated muscles. Evaluation of explanted TA muscle mass revealed that nontreated and CG scaffold-treated tissues (NR: 596.3 ± 55.2 mg, CG: 645.0 ± 49.8 mg), but not CG-PPy-treated tissues (PPy: 683.4 ± 67.5 mg), were significantly lighter than native uninjured muscles (Native: 723.9 ± 50.3 mg, *n* = 24), suggesting a persistent loss of muscle volume. CG-PPy scaffold-treated muscles were significantly heavier than nontreated tissues although this is likely due to the continued presence of residual PPy.

### 3.4 Histological Analysis

**Figure 4:**
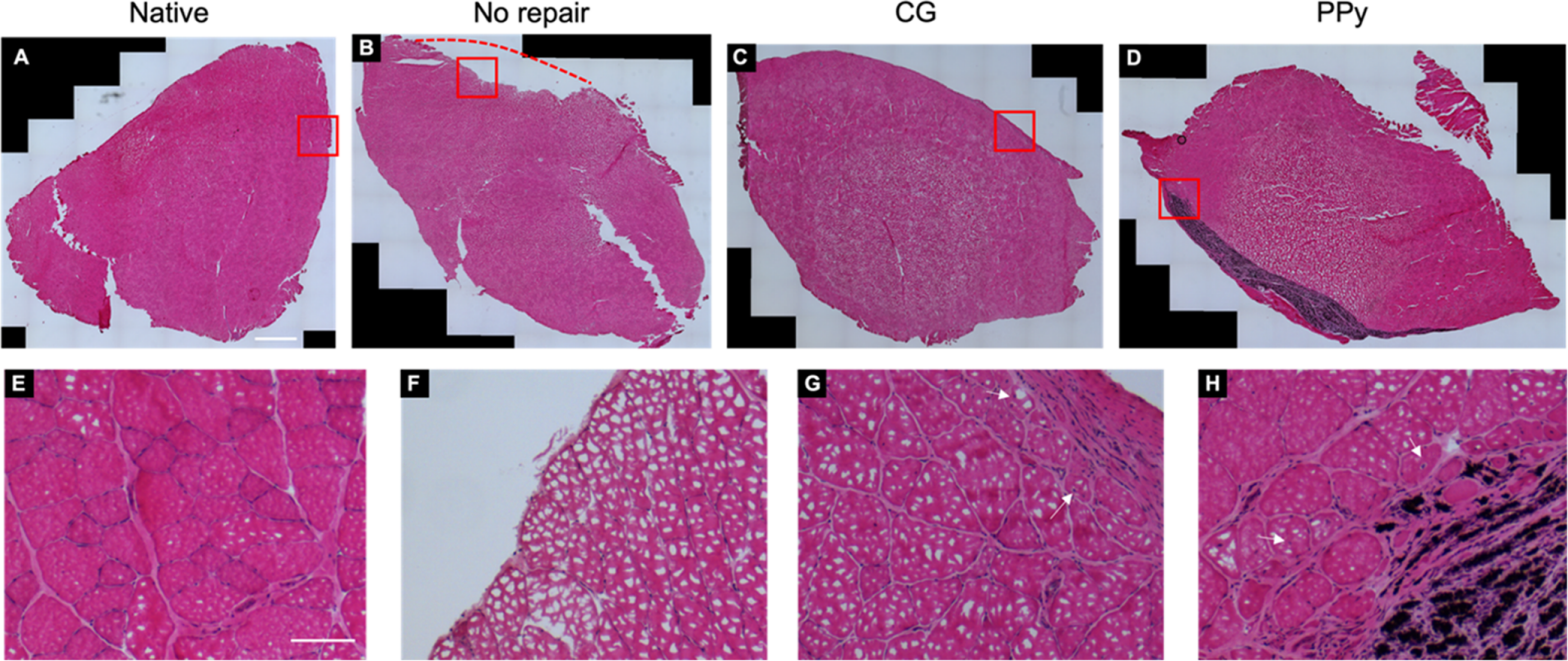
Histological analysis of TA muscles at 12 weeks postinjury. (A) H&E images of TA muscle cross sections for uninjured native tissue, (B) no repair, (C) CG scaffold, and (D) PPy-doped scaffold experimental groups at 12 weeks post-VML. *Dashed red line* indicates the concave VML defect area that remained due to limited tissue regeneration in the no repair group. *Red squares* denote regions of magnified images (E–H). (E) Magnified views of native tissue, (F) no repair, (G) the CG scaffold, and (H) the CG-PPy scaffold experimental groups. *Arrows* indicate myofibers with centrally located nuclei. Scale bars: 1 mm (top); 100 μm (bottom).

After functional testing, explanted muscles were frozen and embedded for histological and immunohistochemical analyses. Muscles explanted from three animals per experimental group were transversely sectioned and underwent H&E staining as well as picrosirius red staining to analyze muscle morphology and fibrotic response following VML injury. Native tissue sections showed an organized network of muscle fibers with nuclei along the periphery of the myofibers characteristic of healthy muscle (Figure 4). As observed from the gross tissue morphology, the VML defect area was easily identified in NR muscles as a clear concave absence of tissue volume with a thin layer of collagenous tissue present, as shown by picrosirius red staining (Figure S2). Moreover, there was a distinct lack of myofibers with centrally located nuclei, a hallmark of regenerating muscle, suggesting limited muscle repair. In contrast, scaffold-treated muscles showed myofibers with centrally located nuclei along the material–tissue interface, potentially indicating myogenesis, although this interpretation is complicated by the presence of freezing artifacts introduced during tissue processing. Closer inspection of CG scaffold-treated muscles revealed a thin layer of connective tissue at the site of material implantation, as indicated by pink fibers, dispersed with cells. Picrosirius red staining confirmed that this connective tissue layer contained collagen. Together, these data indicate that while nonconductive scaffolds supported some level of myogenesis, the scaffold alone could not completely halt the fibrotic response characteristic of VML. Similarly, CG-PPy-treated muscles possessed evidence of regenerating myofibers with centrally located nuclei and a layer of connective tissue at the implantation site although similarly these slides also contained some freezing artifacts. H&E staining also showed that cells infiltrated within the residual PPy particles, suggesting a persistent cellular response and tissue remodeling. Picrosirius red staining indicated significant collagen deposition within the defect site in CG-PPy-treated muscles.

### 3.5 Characterization of Regenerating Muscle Fiber Cross-Sectional Area

**Figure 5:**
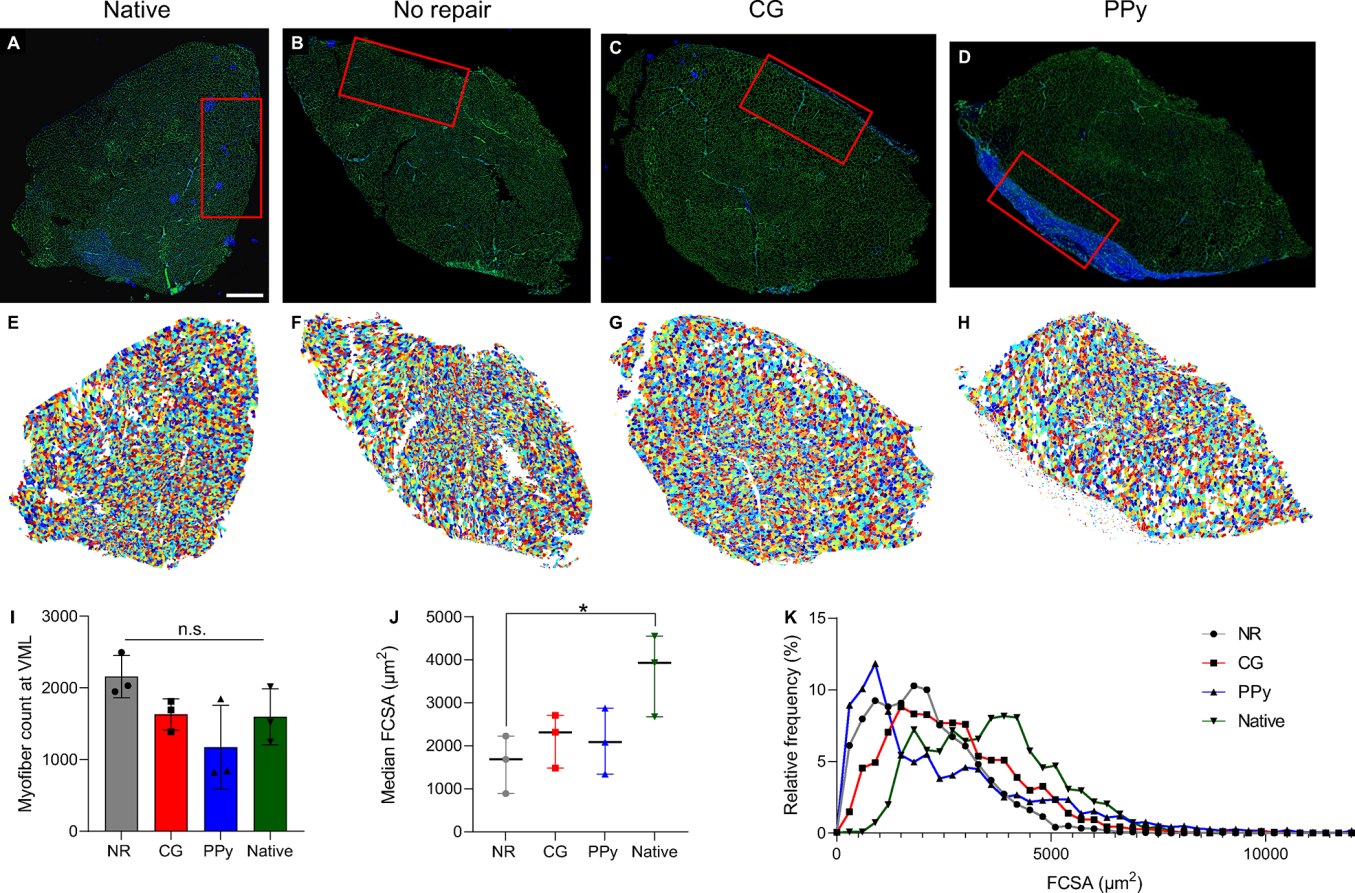
Myofiber cross-sectional area is significantly reduced in nontreated muscle tissues. (A) Representative laminin-stained sections of TA muscles for native uninjured control, (B) no repair, (C) nonconductive CG scaffolds, and (D) conductive CG-PPy scaffold-treated muscles at 12 weeks post-VML. *Red rectangles* denote regions of VML injury used for FCSA analysis. (E, H) Colorized outputs from SMASH software with each color representing individually segmented myofibers corresponding to (A, D), respectively. (I) Muscle fiber count at the VML injury was not statistically different from native muscle across experimental groups. (J) Median fiber cross-sectional area (FCSA) was significantly reduced in NR muscles. (K) FCSA relative frequency curves show a leftward shift toward smaller muscle fibers compared to uninjured muscle regardless of treatment type. Data are presented as Mean ± SD while panel (J) data are presented as the median (line: median) with interquartile range (whiskers). *n*.*s*.: no statistically significant differences. *n* = 3 muscles per experimental group. Scale bar: 1 mm.

To characterize the extent of muscle repair more thoroughly, tissue sections were stained for the ECM protein laminin to visualize individual muscle fibers. Semiautomatic muscle analysis using segmentation of histology (SMASH) software was then used to quantify fiber cross-sectional area (FCSA), the number of muscle fibers, and minimum Feret diameter.^30^ Analysis of the total number of myofibers showed a reduction in fiber counts for all experimental groups compared to native muscles although these results were not statistically significant (NR: 7858 ± 1420; CG: 8077 ± 1235; PPy: 7373 ± 434; Native: 10614 ± 852; Figure S3). The analysis was then repeated for muscle fibers at the location of VML injury, a 4 mm × 2 mm region at the tissue–injury interface, to evaluate regenerating muscle myofibers. Similarly, the number of myofibers at the VML injury site was not statistically different across experimental groups (Figure 5). Assessment of median FCSA and minimum Feret diameter (Figure S4) revealed that myofiber size was significantly reduced in nontreated muscles (1511, 607, and 3031 μm^2^; reported as median, first, and third quartiles) compared to native tissue (3397, 1896, and 5247 μm^2^), indicating muscle atrophy. In contrast, CG and CG-PPy scaffold-treated muscles possessed statistically similar median FCSA (CG: 2080, 778, 3847 μm^2^; PPy: 1617, 603, 4137 μm^2^) compared with uninjured muscles, further corroborating the improved functional outcomes observed. Characterization of FCSA at the VML site also revealed that muscle fibers were generally smaller in experimental tissues compared to native uninjured tissues. This trend is apparent by the increased frequency of smaller diameter fibers and decreased presence of larger muscle fibers compared to fibers in native tissues.

### 3.6 Evaluation of Long-Term Macrophage Polarization

**Figure 6:**
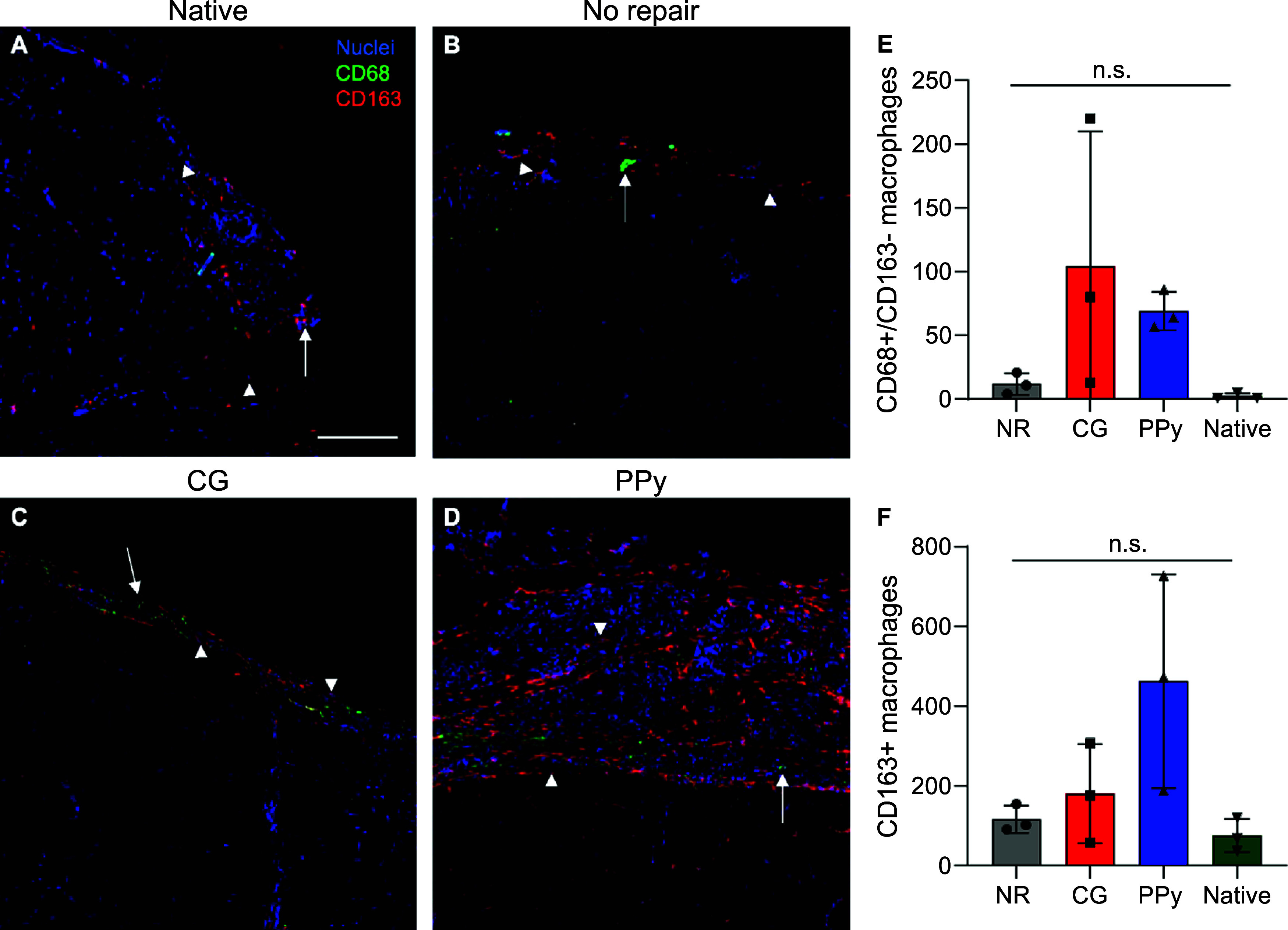
Scaffold-treated muscles show elevated macrophage infiltration at VML injury compared to native muscle. (A–D) Representative images of macrophage infiltration at 12 weeks post-VML. Images were taken at the site of VML injury and stained for CD68 (*green*, arrow) and CD163 (*red*, arrowhead). (E) CD68^+^/CD163^–^ M1 macrophages remained elevated in CG and CG-PPy scaffold-treated muscles, indicating a prolonged wound healing response although these results were not statistically significant. (F) Quantification of CD163^+^ cells indicative of M2 macrophages showed no statistically significant differences across the experimental groups. Units for *y*-axes in panels (E, F): Macrophages per field of view. Data presented as Mean ± SD. *n*.*s*.: no statistically significant differences. *n* = 3 muscles per experimental group. Scale bar: 200 μm.

For minor muscle injuries, a pro-inflammatory immune response is activated within the first few days following injury that is gradually attenuated to allow for myoblast proliferation and differentiation into mature skeletal muscle. However, in the context of VML, a persistent immune response mediated by inflammatory cells such as macrophages results in excessive myofibroblast activation, minimal myogenesis, and limited functional repair.^3^ To more probe how implanted scaffolds impacted the immune response following VML injury, macrophage infiltration and polarization were evaluated. Macrophage presence within the VML injury was characterized using CD68, a pan macrophage marker, and CD163, a marker of alternatively activated M2 macrophages (Figure 6).^32^ The number of classically activated M1 pro-inflammatory macrophages (CD68^+^/CD163^–^) remained elevated in CG and CG-PPy scaffold-treated muscles compared to contralateral control muscles although these results were not statistically significant. The continued presence of M1 macrophages may indicate a persistent inflammatory response.^33,34^ Interestingly, the quantity of CD68^+^/CD163^–^ macrophages within nonrepaired muscles was most similar to native muscle tissues, suggesting that other inflammatory cell types may be the primary drivers of chronic inflammation characteristic of VML. The expression of CD163, indicative of alternatively activated M2 macrophages, was not statistically different across experimental groups at 12 weeks postinjury although their numbers remained elevated in scaffold-treated tissues.^35−37^

### 3.7 Assessment of Vascularization

**Figure 7:**
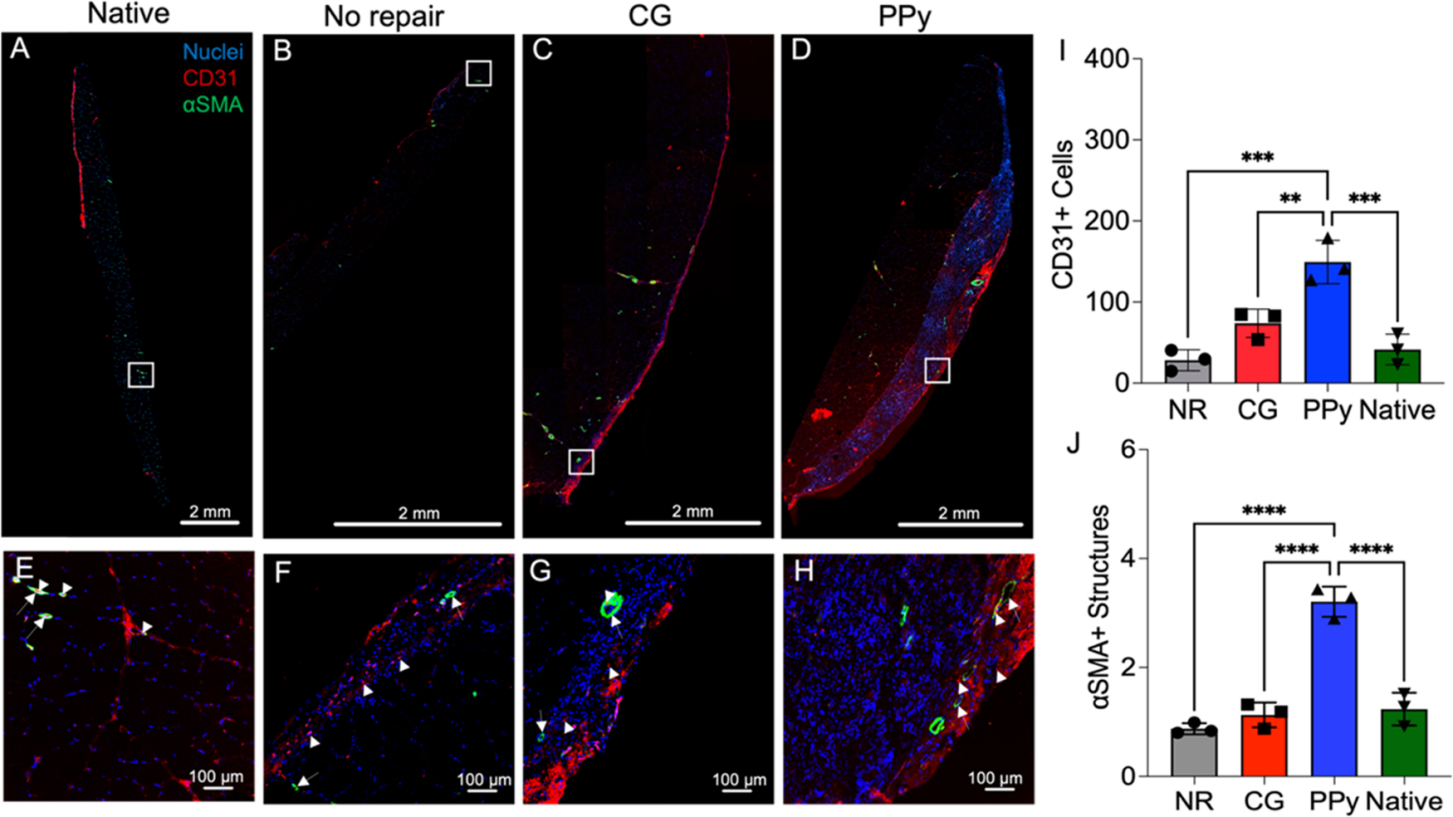
Conductive scaffolds support higher levels of neovascularization at 12 weeks post-VML. (A–D) Representative images of vascular staining at 12 weeks post-VML. Images were taken at and around the defect site showing CD31^+^ cells (*red*, arrowhead) and αSMA^+^ structures (*green*, arrow) within the region of interest. (E–H) Magnified images of muscle defect areas in (E) native, (F) no repair, (G) CG scaffold, and (H) CG-PPy scaffold experimental groups. (I) CD31^+^ cell counts were significantly elevated in CG-PPy scaffold-treated muscles. (J) The number of αSMA^+^ structures was significantly increased in CG-PPy scaffold-treated muscles compared to all other experimental groups. Units for *y*-axes in panels (I, J): Cells/structures per field of view. Data presented as Mean ± SD. ** *P* < 0.01, *** *P* < 0.001, **** *P* < 0.0001. *n* = 3 muscles per experimental group. Scale bars: 2 mm (A–D) and 100 μm (E–H).

Vascularization of muscle samples 12 weeks post-VML was evaluated by using IHC staining. The presence of endothelial cells was examined through CD31 staining, while larger vessels were identified by simultaneous positive detection of CD31 and α smooth muscle actin (αSMA), which serves as a marker for pericytes. Comparing levels of CD31 and αSMA in the experimental groups to those in native muscle tissue, CG-PPy scaffold-treated muscles exhibited significantly higher levels of both CD31^+^ cells and αSMA structures (Figure 7).

### 3.8 Assessment of Muscle Innervation

**Figure 8:**
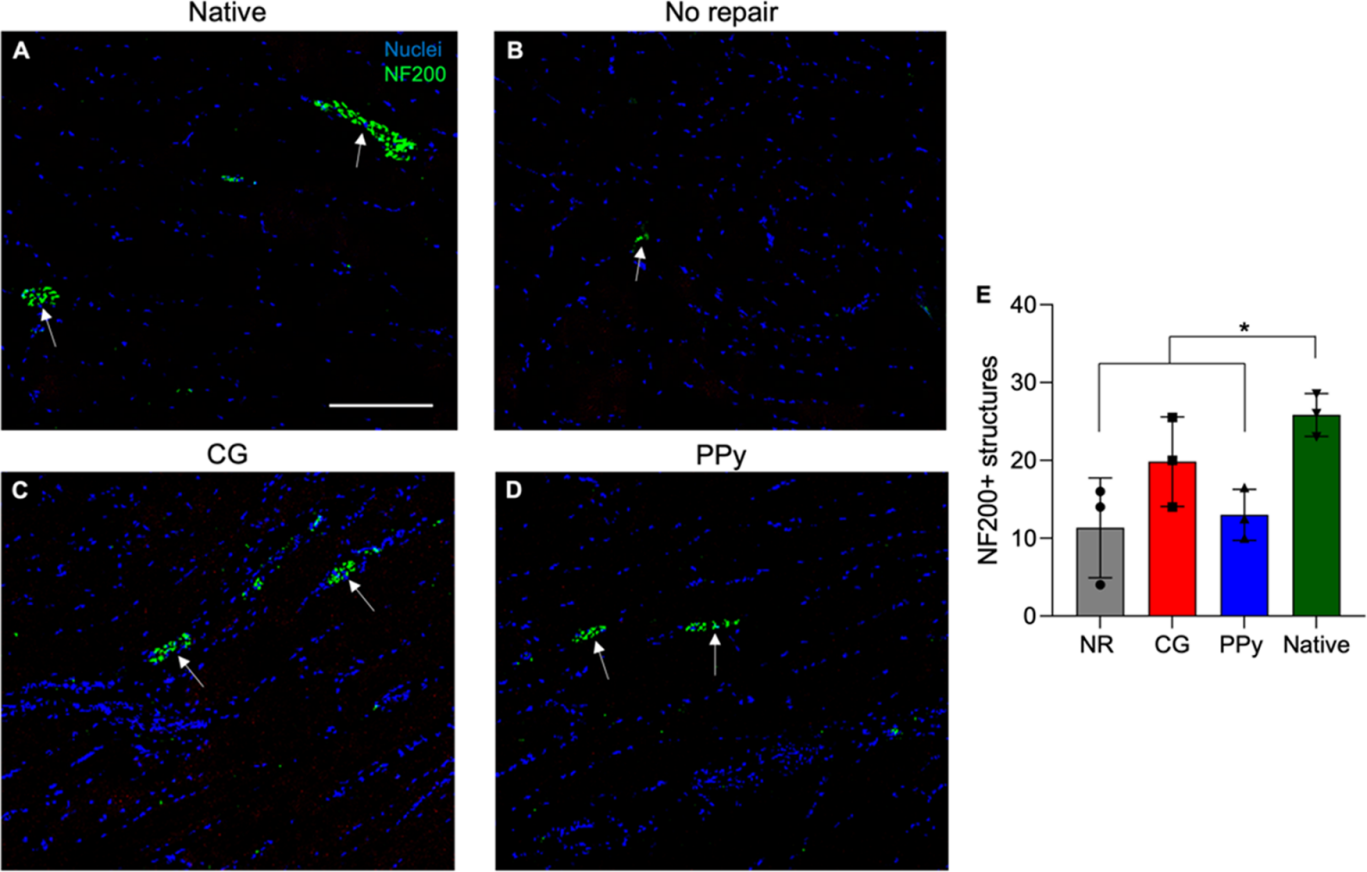
Muscle innervation is significantly reduced in nontreated muscles at 12 weeks post-VML. (A–D) Representative images of neurofilament staining at 12 weeks post-VML. NF200 structures (*green*, arrow) were quantified from images across longitudinal muscle sections. (E) Quantification of NF200 structures revealed that CG scaffold-treated tissues possess statistically similar levels of peripheral nerves compared with native muscle. Units for *y*-axis in panel (E): structures per field of view. Data are presented as Mean ± SD. * *P* < 0.05. *n* = 3 muscles per experimental group. Scale bar: 200 μm.

In addition to evaluating macrophage and vascular cell infiltration, IHC staining of neurofilament 200 (NF200) was used to probe for innervation at 12 weeks post-VML. Rapid skeletal muscle innervation following injury is necessary to facilitate functional recovery, while also limiting prolonged denervation and muscle atrophy. NF200 structures localized toward the middle belly of the TA muscle and were generally observed in clusters surrounding muscle fibers (Figure 8). The presence of NF200 was significantly reduced in NR and CG-PPy scaffold-treated muscles compared to native tissues (NR: *P* = 0.025, PPy: *P* = 0.046), indicating limited innervation post-VML. In contrast, CG scaffold-treated tissues showed higher levels of NF200 structures that were similar to native muscle (CG: *P* = 0.467), corroborating the observed improved functional muscle contraction response to electrical stimulation.

## 4 Discussion

The ideal therapeutic for VML injuries should facilitate restoration of muscle volume, complete with vasculature and innervation, and recovery of tissue function to preinjury levels. While recent advancements in biomaterial design and surgical techniques have shown promise for restoring muscle function in preclinical animal models, they remain limited in their ability to facilitate consistent clinical outcomes. As a result, a rigorous analysis of how material biophysical properties influence the body’s endogenous wound-healing response is required to inform subsequent biomaterial design. Previous work has highlighted that material anisotropy and bioelectrical cues are important regulators of myoblast proliferation and maturation *in vitro*,^38,39^ but it remains unclear how important these factors are in supporting endogenous cell-mediated repair *in vivo*. To address this knowledge gap, we explored how collagen scaffold anisotropy and conductivity impacted skeletal muscle wound healing in a biologically relevant VML injury model.

We chose collagen as our material backbone due to its abundance as a component of the skeletal muscle ECM and previous use as a biomaterial platform for muscle tissue engineering.^22,40,41^ Additionally, collagen-glycosaminoglycan (CG) scaffolds have been widely used across a range of different tissue engineering applications and are clinically approved for the treatment of skin and peripheral nerve defects.^42^ To mimic the high level of organization observed in skeletal muscle, we employed a directional lyophilization technique, using a thermally mismatched mold. The discontinuity in thermal properties within the mold allows for uniaxial heat transfer during freezing which results in the alignment of ice crystals and anisotropy of the collagen struts.^23^ All scaffolds were fabricated at a freezing temperature of −10 °C and a cooling rate of 1 °C/min, which has been shown to support aligned collagen struts, cell proliferation, and organized differentiation of myoblasts.^18,23,43,44^ It is also important to note that naturally derived biomaterial systems are typically limited by their suboptimal mechanical properties and are degraded rapidly by matrix metalloproteinases (MMPs). As a result, we dehydrothermally and chemically cross-linked the collagen and chondroitin sulfate within the scaffolds to increase resistance to enzymatic degradation and improve material mechanics.^45^

In an effort to simulate skeletal muscle’s inherited electrically responsive nature, we aimed to include a conductive moiety within our aligned CG scaffolds. The use of conductive polymers has been explored across a range of electrically excitable cell types and has been shown to facilitate improved cell adhesion, proliferation,^46,47^ and differentiation.^48,49^ The most commonly used conductive polymers in biomaterial systems include polypyrrole (PPy),^50^ polyaniline (PANI),^51^ and poly(3,4-ethylenedioxythiophene) (PEDOT).^52^ Previous work showed that PPy in particular can be easily synthesized and incorporated into tissue engineering scaffolds, resulting in increased electrical conductivity.^53−55^ Additionally, we previously showed that CG-PPy scaffolds supported myoblast growth, organization, and differentiation *in vitro*.^18^ Previous work has also shown that PPy films result in minimal tissue toxicity and immune tissue response when implanted in a variety of animal models.^56−58^ As a result, a logical extension of our previous work was to evaluate how CG scaffolds (with or without conductive PPy) support *in vivo* skeletal muscle wound healing.

Numerous different *in vivo* VML injury models have been evaluated in the literature with the majority focusing on the repair of rodent limb muscles.^31,59−61^ These models are beneficial because they provide a reasonable correlate to common civilian extremity trauma and combat-related injuries.^62^ In this study, biomaterial efficacy was evaluated in a tibialis anterior (TA) model of the VML. CG scaffolds could be easily manipulated and cut to the dimensions of the defect at the time of surgery to ensure complete defect coverage (Figure 1). Additionally, the shape of the scaffolds is only restricted by the geometry of the freeze-drying mold, and as a result, scaffolds can be fabricated to treat a variety of complex and irregular injuries. Our results showed that both nonconductive CG scaffolds and conductive CG-PPy scaffolds facilitated improved functional muscle recovery as measured by *in vivo* maximum force generation compared to nontreated muscle (NR) at 12 weeks post-VML (Figure 2). Moreover, this trend was persistent when muscle force was normalized to baseline values, indicating that functional recovery was not related to animal growth. It is important to note that synergistic muscles, extensor digitorum longus (EDL) and extensor hallucis longus (EHL), were surgically ablated to avoid compensatory hypertrophy following injury. Removal of these muscles results in an approximate 20% reduction in torque generation in the anterior compartment, and as such, comparison of normalized torque postinjury would be theoretically limited to ∼80 N mm kg^–1^ (104.5 N mm kg^–1^ average at baseline). While maximal isometric contraction is an important metric to assess functional muscle recovery, the restoration of submaximal muscle force is also an important clinical outcome to enable precise control of motor function. Muscle force remained statistically similar at lower stimulation frequencies (10–50 Hz) regardless of treatment type, suggesting that biomaterial design can be further optimized to improve functional outcomes. However, scaffold-treated groups produced superior muscle force at moderate frequencies (100–150 Hz) indicating that collagen scaffolds supported improved muscle function and control. Interestingly, the nonconductive scaffolds displayed accelerated functional recovery compared to the NR animals as early as week 8. While this phenomenon invites further investigation, this could potentially be due to more rapid muscle cell infiltration and organization that is somewhat inhibited by residual PPy in the conductive scaffold group. This could also be explained by the higher neurofilament expression in the nonconductive scaffold group (Figure 8), considering motor unit recruitment is critical to force transmission and motor neurons activate muscle contraction through innervating muscle fibers.

To elucidate the differences in cellular response to therapeutic intervention, experimental and contralateral control TA muscles were surgically explanted and flash frozen for histological and immunohistochemical analyses. Unfortunately, freeze artifacts were observed in some muscle sections as circular gaps within muscle fibers, and future work will look to improve freezing methods. Hematoxylin and eosin (H&E) staining revealed a thin layer of regenerating muscle at the material–tissue interface, characterized by small, disorganized muscle fibers with centrally located nuclei (Figure 4), a finding not observed in nontreated tissues. These findings corroborate our functional testing data and suggest that both scaffold groups facilitated superior myogenesis, which ultimately leads to improved muscle function. CG-PPy scaffold-treated muscles also showed residual conductive particles at the location of implantation. We have previously determined that PPy particles are approximately 500 nm in diameter and thus are not likely to be phagocytosed by cells. Our findings also reflect previously published work where an electrically responsive PPy-chitosan hydrogel was injected into the peri-infarct region of a rat heart 1 week after myocardial infarction.^63^ In this study, the researchers observed that the PPy-chitosan hydrogel was still visible in the peri-infarct space and embedded in the repairing cardiac tissue of Masson trichrome-stained sections 8 weeks after injection. However, despite the persistence of the conductive moiety at the injury site, PPy-chitosan gels facilitated increased transverse activation velocity and load-dependent ejection fraction fractional shortening in comparison with saline or chitosan injections.^63^ Together these findings suggest that while PPy particles may remain at the site of injury 12 weeks postinjury, CG-PPy scaffold implantation may still support improved muscle function. Future work may look to improve the cellular breakdown of conductive polymers by incorporating enzymatically degradable motifs although such modification would likely decrease electrical properties.

It is important to note that despite evidence of myogenesis in scaffold-treated groups, a significant amount of fibrotic tissue remained (Figure S2), and all experimental tissues had significantly reduced muscle volume compared to native TA muscles (Figure 3). Previous work by our group and others have described the phenomenon of functional fibrosis in which some improvement in muscle function is observed, but very little *de novo* myogenesis occurs.^64−66^ In this paradigm, the therapeutic mainly functions by providing passive mechanical force transmission along the remaining intact muscle, approaching the theoretical maximal isometric contraction. In an effort to limit the development of functional fibrosis, the inclusion of myogenic cells within the regenerative therapeutic is potentially necessary to restore muscle volume and function.^31,64,67^ A meta-analysis exploring regenerative therapies following VML supports this hypothesis, stating that currently the most effective treatment for functional repair of skeletal muscle combines engineered biomaterials and seeded myogenic cells.^67^ These findings are further supported by our group where a decellularized porcine bladder extracellular matrix (BAM) resulted in minimal functional muscle recovery and extensive fibrosis when implanted into a VML injury.^64^ Conversely, when the BAM was reseeded with myogenic cells, substantial *de novo* myogenesis was observed, leading to superior functional recovery in numerous animal models.^25,31,59^

Although previous work highlights the importance of a cellular component to facilitate robust muscle regeneration, we chose to deploy the scaffolds acellularly due to the reduced regulatory challenges associated with these materials. Moreover, acellular CG scaffolds produced via freeze-drying are currently clinically approved for the treatment of skin and peripheral nerve injuries.^21,42^ While our scaffold system did not appear to facilitate extensive myogenesis, scaffold-treated muscles showed some evidence of regenerating myofibers, as well as superior muscle force at submaximal electrical stimulation, that were not observed in NR tissues. We believe that these enhanced regenerative outcomes are due to improved cell infiltration and remodeling at the site of VML. Freeze-dried CG scaffolds possess an open, interconnected pore microstructure (pore diameter ∼150 μm) that allows rapid cellular infiltration while facilitating nutrient and waste transport.^18^ In contrast, traditional hydrogel systems contain nanometer-scale mesh sizes that are orders of magnitude smaller^23,42,68^ and inhibit these processes. In this initial study, cell and tissue organization was only characterized at 12 weeks postinjury, when scaffolds appeared to have fully degraded. Future work should assess cell infiltration, including the presence of pro-regenerative cells such as Pax7+ satellite cells, into scaffolds at early time points postinjury to better evaluate endogenous repair. Despite this limitation, our findings highlight the use of CG scaffolds as potential therapeutics for VML injuries (Table 1).

**Table 1:** Summary of Experimental Findings across Treatment Groups^a^

treatment groups	no repair	nonconductive CG scaffold	conductive CG-PPy scaffold	native tissue	figure and table
sample size (*n*)	8	8	8	24	Figure 1
defect weight (mg)	105.3 ± 11.6	114.6 ± 14.2	116.0 ± 7.8	---	Figure 1F
baseline max torque (N-mm kg^–1^)	105.2 ± 6.6	103.1 ± 4.1	105.1 ± 4.5	---	Figure 1G
4 wk max torque (N-mm kg^–1^)	49.5 ± 5.7	54.8 ± 3.8	55.3 ± 7.8	---	Figure 2A
8 wk max torque (N-mm kg^–1^)	55.0 ± 5.9	64.7 ± 6.2	61.1 ± 8.5	---	Figure 2B
12 wk max torque (N-mm kg^–1^)	54.0 ± 5.2	66.3 ± 7.2	65.1 ± 6.0	---	Figure 2C
peak isometric torque (% of baseline @ 12 wk)	51.6 ± 6.4	64.5 ± 8.2	62.0 ± 5.7	---	Figure 2D
explant muscle weight (mg)	*596.3 ± 55.2*	*645.0 ± 49.8*	683.4 ± 67.5	723.9 ± 50.3	Figure 3E
muscle weight/body weight	*0.00142 ± 0.00012*	*0.00145 ± 0.00012*	*0.00152 ± 0.00009*	0.00165 *±* 0.00007	Figure 3F
fiber count at VML site	2158 ± 294	1630 ± 219	1171 ± 586	1596 ± 390	Figure 5I
Median FCSA (μm^2^) at VML site	*1511, 607, 3031*	2080, 778, 3847	1617, 603, 4137	3397, 1896, 5247	Figure 5J
CD68^+^/CD163^–^ surrounding injury	12.0 ± 8.5	104.3 ± 105.6	69.0 ± 15.1	2.3 ± 2.3	Figure 6E
CD163^+^ surrounding injury	116.0 ± 34.2	181.7 ± 124.5	462.3 ± 268.6	75.0 ± 41.9	Figure 6F
CD31^+^ cells	28.3 ± 12.9	73.8 ± 17.5	*149.4 ± 26.7*	41.5 ± 18.9	Figure 7I
αSMA^+^ structures	0.9 ± 0.1	1.1 ± 0.2	*3.2 ± 0.3*	1.2 ± 0.3	Figure 7J
NF200^+^ structures	*11.3 ± 6.4*	19.8 ± 5.8	*13.0 ± 3.3*	25.8 ± 2.8	Figure 8E

a Values are presented as mean ± standard deviation except for median minimum FCSA, which is reported as the median, first, and third quartiles. Values denoted with the bolded lettering are significantly different (*P* < 0.05) from the no repair group, while values with italic lettering are significantly different (*P* < 0.05) from the native tissue contralateral control muscles. FCSA, fiber cross sectional area; NF200, Neurofilament 200; ---, analysis not applicable to native tissue contralateral control.

To further characterize the extent of skeletal muscle repair, SMASH analysis was used to quantify the total myofiber number and fiber cross-sectional area (FCSA) (Figure S3). Analysis of myofiber counts showed that the number of muscle fibers was marginally reduced compared to native muscle tissues, indicating suboptimal recovery of muscle volume. This result further supports the claim that a cellular component is likely necessary to augment endogenous repair mechanisms to recover adequate muscle mass and function. Additional characterization of muscle FCSA at the VML injury revealed that median fiber size was significantly reduced in NR muscles compared with native tissue (Figure 5). In contrast, median myofiber FCSA within scaffold-treated tissues was not statistically different from uninjured tissues, indicating that scaffolds facilitated superior muscle recovery. These findings support our functional data suggesting that the improvements observed in muscle torque production are related to improved cellular repair and wound healing, as myogenesis was not present in nontreated muscles. Despite improvements in median muscle FCSA, scaffold-treated tissues still displayed a leftward shift toward smaller muscle fibers compared to uninjured muscle. These findings suggests that while collagen scaffolds may support improved muscle function, they remain limited in their ability to facilitate extensive myogenesis. As a result, future work could look to improve repair of muscle volume using CG scaffolds by inclusion of myogenic and supporting cells or other bioactive moieties to further augment endogenous repair mechanisms.

VML injuries are characterized by a persistent inflammatory response that limits myogenic cell expansion and instead facilitates myofibroblast activation, ECM deposition, and fibrosis. While many inflammatory cells are involved in skeletal muscle repair, macrophages have been highlighted as important mediators of the wound healing cascade that can either serve as key constituents of pro-regenerative microenvironments or perpetuate chronic inflammation.^35,36^ During normal wound healing, a carefully controlled shift in macrophage polarization from a classically activated M1 “pro-inflammatory” phenotype toward a predominately alternatively activated M2 “pro-regenerative” phenotype allows for robust myoblast expansion followed by differentiation and maturation.^35−37^ However, during VML injuries, macrophages are persistently activated, thereby limiting endogenous repair mechanisms and instead promoting the activation of cells from fibrogenic lineages.^3,69^ By evaluating macrophage infiltration and polarization within the VML injury area, we aimed to assess the scaffold’s ability to support a pro-regenerative microenvironment (Figure 6). The number of M1 (CD68^+^/CD163^–^) and M2 (CD163^+^) macrophages remained elevated in CG-treated muscles (M1: *P* = 0.169; M2: *P* = 0.825) compared to that of native muscle, suggesting a persistent inflammatory response. While not statistically significant, perhaps due to underpowered histological analysis (*n* = 3 animals per group), CG-PPy scaffold-treated muscles also showed an increase in both M1 and M2 macrophages, indicating continual remodeling (M1: *P* = 0.467; M2: *P* = 0.054). Interestingly, nontreated muscles showed macrophage staining profiles most similar to native tissues. Typically, during wound healing, macrophages return to baseline values at approximately 2 weeks postinjury to allow for the repair and remodeling phases. As a result, we hypothesize that the wound healing response reached completion prior to 12 weeks in NR tissues, leading to a reduction in the number of macrophages and the presence of necrotic muscle. Future work would benefit from exploring earlier time points postinjury and assessment of additional cell types involved in aberrant wound healing, such as fibroadipogenic progenitor cells, to gain a more complete picture of the repair process.

After investigating macrophage localization, we next quantified the number of endothelial cells (CD31^+^) and pericytes (αSMA^+^ cells colocalized with CD31^+^ endothelial cells) as a proxy of vascularization (Figure 7). Given the high metabolic needs of skeletal muscle, angiogenesis and revascularization following VML are integral to functional recovery.^70^ Significantly higher numbers of CD31^+^ cells and αSMA^+^ structures were observed in the CG-PPy scaffold-treated muscles compared with the other experimental groups. While other work has shown that PPy materials can enhance vascularization,^71,72^ our observed results may be due to the overall higher level of cellularity in the PPy group. Future work could further improve vascularization by tuning scaffold properties such as glycosaminoglycan content, where previous work incorporating heparin significantly improved angiogenic outcomes.^29,73,74^

While the restoration of muscle volume is essential for improved regenerative outcomes, innervation of repaired tissue is also required for proper function. Electrically responsive polymers have previously been shown to facilitate increased neural stem cell proliferation and differentiation *in vitro* compared to nonconductive substrates.^58,75^ Therefore, we aimed to assess whether scaffolds allowed superior skeletal muscle innervation *in vivo* compared to other experimental groups. Myofibers are innervated at neuromuscular junctions (NMJs) that are often found along the sarcolemma toward the middle of the muscle belly to allow for the effective propagation of action potentials.^76^ As a result, TA muscles were cut in half, sectioned longitudinally, and stained for neurofilament 200 (NF200) to visualize neurons (Figure 8). Only nonconductive CG scaffolds contained statistically similar levels of NF200^+^ structures compared to native muscle. In contrast, no repair and conductive CG-PPy scaffold-treated tissues possessed significantly reduced numbers of NF200^+^ structures. These findings corroborate our functional testing data in which only CG scaffold-treated muscles produced increased isometric torque at submaximal stimulation frequencies of 60 and 80 Hz. We hypothesize that the lack of innervation in CG-PPy scaffold-treated muscle may be due to reduced electrical dopant stability under physiological conditions. Previous work has shown that substrate conductivity can decrease substantially over time reducing the electrical properties of the scaffold.^77^ The findings further indicate that while conductive PPy may facilitate improved cell development *in vitro*, further material optimization is required for *in vivo* efficacy.

## 5 Conclusions

This study aimed to improve the treatment of VML injuries by designing a biomaterial system that mimics biophysical features critical to healthy function, including three-dimensional (3D) anisotropy and electrical excitability. In the present study, we assess the efficacy of a 3D-aligned collagen scaffold platform to repair a biologically relevant TA model of VML injury. Nonconductive CG and electrically conductive CG-PPy scaffolds were prepared by directional lyophilization and surgically implanted into VML defects. Both scaffold-treated groups supported increased functional muscle recovery at 12 weeks postinjury compared to nontreated muscles as measured by *in vivo* electrical stimulation of the peroneal nerve. Subsequent histological analysis of the material–tissue interface showed regenerating muscle fibers in scaffold-treated muscles, indicated by small myofibers with centrally located nuclei, that were not observed in nonrepaired tissues. Further analysis of myofiber size showed that nontreated tissues possessed significantly reduced median FCSA compared to native muscles, indicating impairment of muscle regeneration. Immunohistochemical analyses revealed that scaffold-treated muscles possessed higher numbers of macrophages and conductive scaffold-treated muscles had higher numbers of vascular cells, suggesting a persistent wound healing response that was not observed in nontreated tissues. Finally, nonconductive scaffolds alone possessed statistically similar levels of neurofilament staining compared to native tissue, further corroborating the improved isometric contraction observed. While the addition of PPy largely did not appear to result in improved functional outcomes compared to nonconductive CG scaffolds, our findings illustrate that aligned collagen scaffolds can facilitate improved functional recovery and endogenous muscle repair following VML injury. Future work should look to optimize the synthesis of CG scaffold composites, perhaps through the inclusion of cells and/or degradable conductive polymers, to further augment skeletal muscle regeneration.

## Supporting Information Available

Scaffold mechanical characterization; additional histology and immunohistochemical analysis of repaired muscle (PDF)
