# Supplementary material for: Freeze-Dried Porous Collagen Scaffolds for the Repair of Volumetric Muscle Loss Injuries
Source: ACS Biomater Sci Eng. 2025 Feb 5;11(3):1598–611. doi: 10.1021/acsbiomaterials.4c01601 (PMC11897937; doi:10.1021/acsbiomaterials.4c01601)
Supplement: Supplementary file 1 — ab4c01601_si_001.pdf [file ab4c01601_si_001.pdf]

Supporting Information

Freeze-dried porous collagen scaffolds for the repair of volumetric muscle loss injuries

Ivan M. Basurto[1], Geshani C. Bandara[2], Ryann D. Boudreau[1], Sydney B. Shriver[1], Samir A. Muhammad[1], George J. Christ[1,3*], Steven R. Caliari[1,2*]

[1]Department of Biomedical Engineering, [2]Department of Chemical Engineering, [3]Department of Orthopedic Surgery, University of Virginia [*]Co-corresponding authors

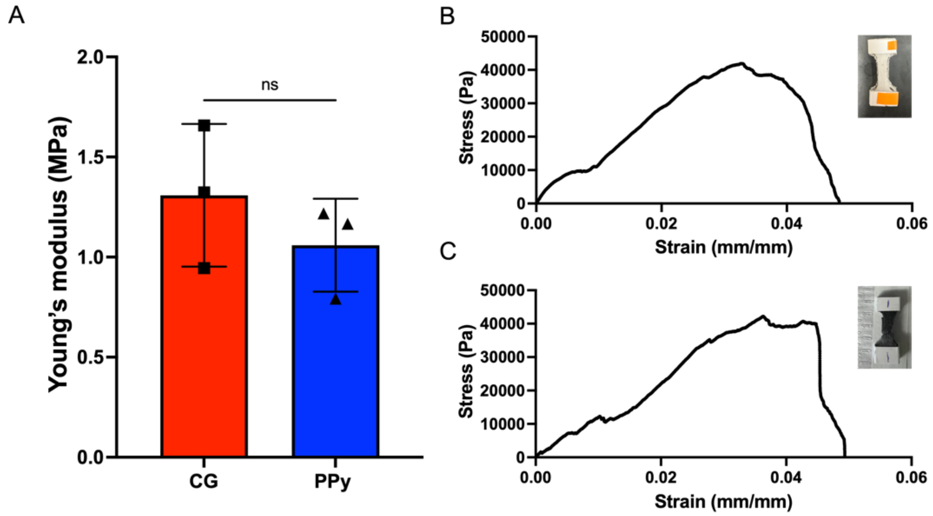

Figure S1: PPy incorporation does not significantly alter scaffold Young's modulus. (A) Young's modulus was measured by tensile testing of dry scaffolds to failure. Representative stress-strain curves for (B) CG and (C) CG-PPy scaffolds. n.s.: no statistically significant differences. *n* = 3 scaffolds per experimental group.

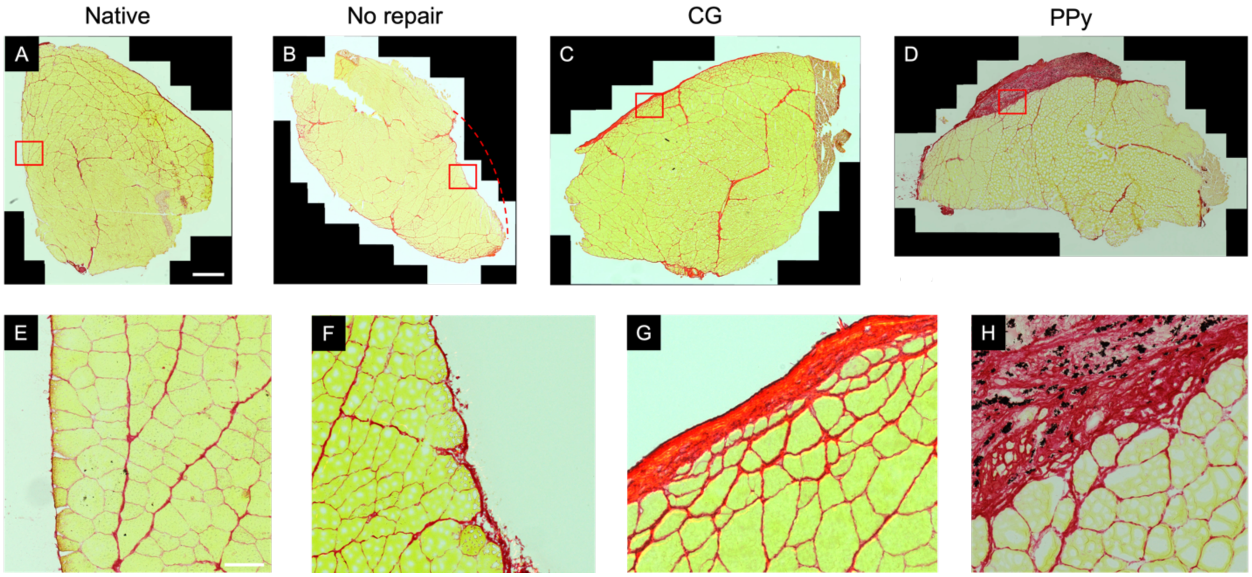

Figure S2: Picrosirius red analysis of TA muscles at 12 weeks post-injury. (A) Picrosirius red images of TA muscle cross-sections for uninjured native tissue, (B) no repair, (C) CG scaffold, and (D) PPy-doped scaffold experimental groups at 12 weeks post-VML. *Dashed red line* indicates the concave VML defect area that remained due to limited tissue regeneration in the no repair group. *Red squares* denote regions of magnified images (E-H). (E) Magnified views of native tissue, (F) no repair, (G) CG scaffold, and (H) CG-PPy scaffold experimental groups. Scale bars: 1 mm (top), 100 µm (bottom).

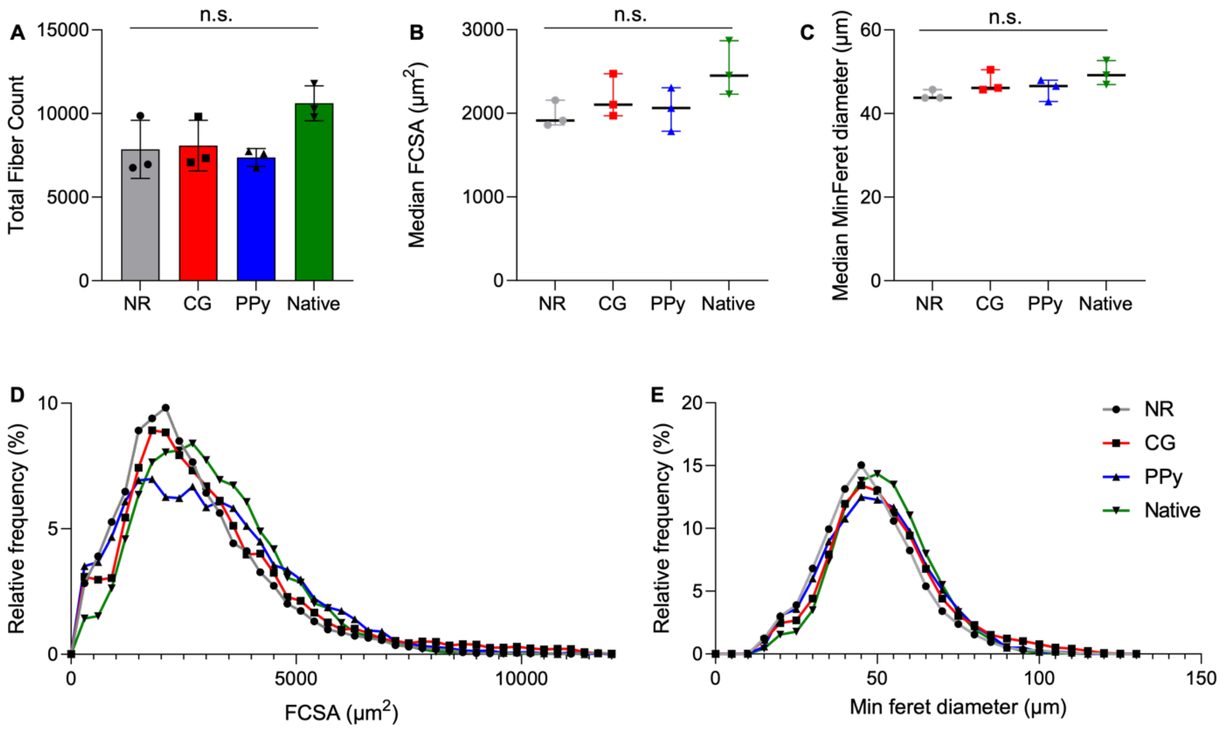

Figure S3: Total myofiber number and cross-sectional area is reduced regardless of treatment type. (A) Total myofiber count was not statistically different from native muscle across experimental groups, although the number of muscle fibers was reduced. (B) Total median fiber cross-sectional area (FCSA) and (C) median minimum Feret diameter were statistically similar across all experimental groups. (D) FCSA and (E) minimum Feret diameter relative frequency curves show similar distributions of myofiber size regardless of treatment type. Data presented as Mean +/- SD while panel (B) and (C) data are presented as the median (line: median) with interquartile range (whiskers). n.s.: no statistically significant differences. n = 3 muscles per experimental group.

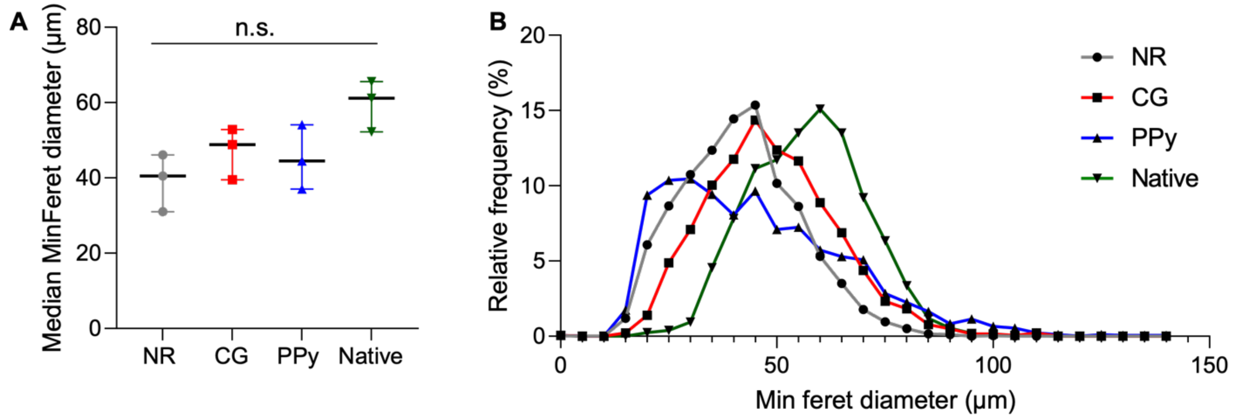

Figure S4: Myofiber minimum Feret diameter is reduced in non-treated muscle tissues at the site of VML injury. (A) Minimum Feret diameter was reduced in NR muscles, although these results were not statistically significant. (B) Minimum Feret diameter relative frequency curves show a similar leftward shift toward smaller muscle fibers across experimental tissues compared to native muscles. Panel (A) data are presented as the median (line: median) with interquartile range (whiskers). n.s.: no statistically significant differences. n = 3 muscles per experimental group.
